# Retraction: Mechanism of Action of the Novel Nickel(II) Complex in Simultaneous Reactivation of the Apoptotic Signaling Networks Against Human Colon Cancer Cells

**DOI:** 10.3389/fphar.2016.00199

**Published:** 2016-06-29

**Authors:** Frontiers in Pharmacology Editorial Office

The Journal and Authors retract the 28 January 2016 article cited above for the following reasons: Following online criticisms of the published paper, an investigation at the journal has confirmed the manipulation and duplication of data, and a level of image processing that is not compliant with the journal’s policies on image data integrity.

Further, a post-publication cross-check screening revealed significant overlap with another manuscript submitted by the authors to another journal. While all manuscripts submitted to Frontiers undergo cross-check screening, the article cited below was in review in parallel with the Frontiers article and thus the overlap was not apparent until publication (Samie et al., 2016).
